# Eosinophilic Granulomatosis Polyangiitis (EGPA) Masquerading as a Mycotic Aneurysm of the Abdominal Aorta: Case Report and Review of Literature

**DOI:** 10.1155/2021/7093607

**Published:** 2021-09-13

**Authors:** Pooja Kumari, Debendra Pattanaik, Claire Williamson

**Affiliations:** ^1^Division of Rheumatology, Department of Medicine, University of Tennessee Health Science Center, Memphis, TN 38163, USA; ^2^Department of Pathology and Laboratory Medicine, University of Tennessee Health Science Center, Memphis, TN, USA

## Abstract

**Introduction:**

Aortic involvement leading to aortitis in eosinophilic granulomatosis polyangiitis (EGPA) is infrequent, and only 2 cases have been reported so far in the literature. Even more so, aortic aneurysm, secondary to EGPA, has never been reported and remains a diagnostic and therapeutic challenge. *Case Presentation*. We present a 63-year-old Caucasian male patient with a prior diagnosis of EGPA presenting with abdominal pain, nausea, and loose stools to the emergency department. Physical examination showed periumbilical tenderness. He had no peripheral eosinophilia but had high C-reactive protein and procalcitonin levels. CT abdomen revealed a mycotic aneurysm involving the infrarenal abdominal aorta. The patient declined surgical repair initially and was treated with IV antibiotics only. Unfortunately, 24 hours later, the aneurysm ruptured, leading to emergent axillofemoral bypass surgery. Surgical biopsy showed aortitis, periaortitis, and active necrotizing vasculitis.

**Conclusion:**

Abdominal aneurysms should be considered a complication of EGPA, and earlier immunosuppressive therapy should be considered to prevent further complications.

## 1. Introduction

Previously called Churg Strauss syndrome (CSS), eosinophilic granulomatosis with polyangiitis (EGPA) is a subtype of antineutrophil cytoplasmic antibody- (ANCA-) associated vasculitis (AAV) [1]. It is characterized by eosinophilic necrotizing inflammation involving medium- and small-size vessels [1]. Involvement of large vessels is sporadic, with only 2 cases being reported presenting as aortitis in the literature. There is no reported case of aortic aneurysm in EGPA. We report here the first case of abdominal aortic aneurysm secondary to EGPA masquerading as a mycotic aneurysm, leading to delay in diagnosis and appropriate treatment.

## 2. Case Presentation

**Figure 1:**
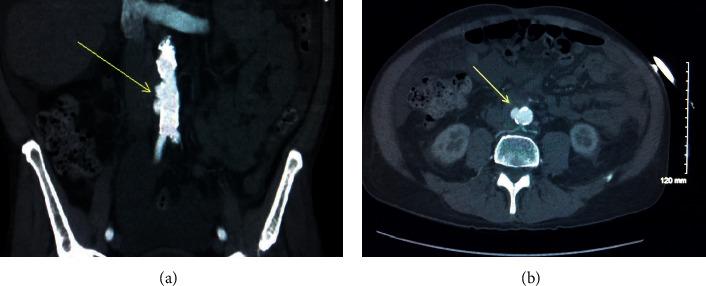
(a and b) presence of multilobulated aneurysm of infrarenal abdominal aorta with aortic wall thickening and subtle fatty stranding; aneurysm is indicated by arrows, on coronal and axial images from CT of the abdomen and pelvis with iodinated contrast.

**Figure 2:**
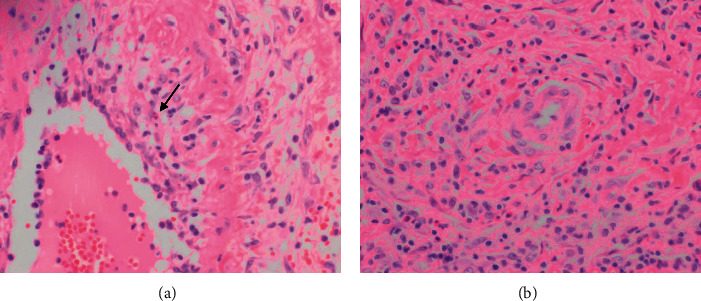
(a) Histopathologic sections of the aorta show vasculitis of small and medium vessels with occasional eosinophils within the vessel wall. (b) Infiltration of the periaortic tissue with eosinophils and other inflammatory cells.

A 63-year-old Caucasian male subject presented to GI clinic in May 2009 with complaints of bloating, constipation, and 30 pounds weight loss for the past 6 months. He denied any abdominal pain, nausea, vomiting, dysphagia, diarrhea, hematochezia, or rash. Past medical history includes allergic rhinitis, chronic sinusitis, hearing loss, psychotic, and paranoid personality disorder. He had an appendectomy before. He denied smoking, alcohol, or any illicit drug use. Physical examination revealed nasal polyps, but the rest of the examination was unremarkable. Complete blood count showed peripheral blood eosinophilia of 53.1% (0.1–8), elevated erythrocyte sedimentation rate (ESR) of 31 mm/hour (0–20), and C-reactive protein (CRP) of 22.7 mg/L (0.0–4.0). Liver function tests (LFTs) showed elevated alkaline phosphatase of 285 U/L (40–140) and elevated gamma-glutamyl transferase (GGT) of 135 U/L (10–75). Antinuclear antibody (ANA), complements, cytoplasmic antineutrophilic antibodies (c-ANCA), proteinase3 (PR3) antibody, and myeloperoxidase (MPO) antibody were all normal. However, perinuclear antineutrophil cytoplasmic antibody (p-ANCA) was positive in low titer: 1 : 80. Stools for ova and parasites were negative but had a positive toxocara antibody. He was treated with ivermectin without any improvement in symptoms. Subsequently, a bone marrow biopsy was done and was negative for any myeloproliferative disorders. Biphasic liver CT showed diffuse portal fibrosis concerning biliary cirrhosis. A duodenal biopsy revealed areas with profound eosinophilic infiltrate (>100 eosinophils/HPF) suggestive of eosinophilic enteritis. A liver biopsy showed multiple hepatic granulomas with central necrosis and extensive eosinophilia. Based on the constellation of symptoms and biopsy findings, he was diagnosed with EGPA. Treatment with high-dose steroids was recommended, but the patient declined immunosuppressive medications due to concern for side effects. He continued to follow with allergy/immunology and rheumatology service and had multiple hospitalizations for asthma flare. He was receiving treatment with on and off corticosteroids for asthma flare and inhalers for asthma. In January 2020, he presented to the emergency department (ED) with a 2-week history of sudden onset sharp and right-sided lumbar back pain, which started after moving a large spare tire. He also reported pain in the umbilical region aggravated by meals and associated with nausea and loose stools. Vital signs were as follows: Blood pressure 129/77 mm of hg, pulse was 79/minute, RR was 12/minute, and temperature was 36.6°C. Physical examination was significant only for periumbilical tenderness. Laboratory tests showed white blood cell count of 14,240/ul (4000–10,000) and differentials of eosinophils 2% (0.1–8), polymorphonuclear cells 82% (38–72), and lymphocytes 8.3% (15–45). Hemoglobin and platelet counts were normal. CRP was 104 mg/L (0.0–5.0) and procalcitonin was 7.4 ng/mL (0.0–0.25). Renal function and urinalysis were normal. The liver function test showed normal transaminases, but alkaline phosphatase was elevated at 217 U/L (40–150). ANCA panel and ANA were negative. IgG4 was minimally elevated at 125 mg/dl (2–96). Blood cultures were negative. CT abdomen and pelvis with and without contrast showed asymmetric fusiform sac measuring 3.6 ×  3.2 cm  along the right lateral and anterior margin of the abdominal aorta at the level of L4. There was aortic wall calcification along the right lateral margin and hazy wall thickening of the aneurysm sac with surrounding stranding and haziness. These are suggestive of mycotic aneurysms (Figures 1(a) and 1(b)). He was admitted to vascular surgery service. Infectious disease and rheumatology services were consulted. He was started on broad spectrum IV antibiotics, but he refused any surgical intervention for the aneurysm. The following day, the patient had worsening abdominal pain and a repeat CT abdomen with contrast showed localized areas of contrast bulging in the right side of the infrarenal abdominal aorta, projecting into the periaortic soft tissue thickening. These contrast projections extended beyond the calcified wall of the aorta and were contained within the soft tissue thickening; findings were consistent with confined aortic rupture associated with periaortic inflammatory thickening. He was taken to the operating room for an emergent exploratory laparotomy. The infrarenal aorta was resected and a right axillobifemoral bypass was performed with an 8 mm polytetrafluoroethylene (PTFE) graft. Aortic wall tissue was sent for histopathology analysis. Aortic wall pathology showed markedly inflamed, hemorrhagic, and partially necrotic periaortic and scant aortic tissue infiltration of plasma cells, lymphocytes, and neutrophils and histiocytes with occasional eosinophils within the vessel wall. It also showed eosinophilic vasculitis of mostly small and rarely medium vessels with rare fibrinoid necrosis (Figures 2(a) and 2(b)). No granuloma was identified. Gram stain and Periodic Acid Schiff Stain (PAS) stain for fungus were negative. The final pathologic diagnosis was aortitis, periaortitis, and necrotizing vasculitis suggestive of EGPA. After multiple other postsurgical complications, he had a prolonged and difficult recovery in the surgical intensive care unit. After a month-long recovery period, he was offered further immunosuppressive therapy, which he continues to decline.

## 3. Discussion

We presented a case of long-standing EGPA, presenting to the emergency department with abdominal pain and CT abdomen findings of a mycotic aneurysm involving infrarenal abdominal aorta. Unfortunately, the aneurysm ruptured, and emergent surgery was performed. The resected aortic tissue showed aortitis, periaortitis, and necrotizing vasculitis. Postoperatively patient had a slow prolonged recovery, and he continued to decline further immunosuppressive treatment.

Granulomatosis polyangiitis (GPA), microscopic polyangiitis (MPA), and EGPA are classified as AAV involving small- and medium-size vessels [1]. Several case series have reported the involvement of large vessels in patients with GPA and MPA [2–4]. However, large vessel involvement including the aorta resulting in aortitis has been reported only in 2 cases of EGPA who were newly diagnosed but none had aortic aneurysm [5, 6]. Aortic involvement in our case happened almost ten years after the initial diagnosis and resulted in aneurysm formation, unlike in previously 2 reported cases. The pathogenesis of ANCA-associated large vessel disease is unknown. It is believed that ANCAs contribute to the pathogenesis of small and medium vessel vasculitis [7]. ANCAs may be involved in the pathogenesis of large vessel vasculitis as well [3]. Our patient only had low titer p-ANCA positivity on initial presentation ten years ago but was negative afterward including the MPO antibody. One study reported a role of vasovasoritis, e.g., inflammation of small vessels feeding the large arteries, and leading to the development of the large vessel vasculitis [2]. Another case reported only intimal involvement without any involvement of media and adventitia [4]. However, histopathologic analysis of 5 cases revealed transmural aortitis [3]. Based on this evidence, it is possible that intimal injury occurs as the initial component of ANCA-associated large vessel disease and slowly involves the entire vessel wall [3].

Infected aneurysms, otherwise known as “mycotic” aneurysms, and inflammatory aneurysms account for a minority of cases of abdominal aortic aneurysms [8]. Inflammatory aortic aneurysms are usually seen with giant cell arteritis and Takayasu arteritis but are rarely reported with Bechet's disease, sarcoidosis, Kawasaki disease, rheumatoid arthritis, ankylosing spondylitis, Systemic Lupus Erythematosus, Cogan's syndrome, IgG4 disease, granulomatous, and microscopic polyangiitis [9]. Mycotic aortic aneurysms are rare but potentially life-threatening due to the elevated risk of rupture. It can occur due to direct bacterial inoculation into the arterial wall at the time of vascular injury or from hematogenous spread of systemic infection, contiguous involvement of vessel wall from an adjacent source or due to septic emboli from the heart. Mycotic and inflammatory aneurysms both present with similar nonspecific symptoms, e.g., abdominal or back pain, weight loss, and low grade fever, and are difficult to diagnose [8]. There are few nonspecific imaging findings that can help distinguish mycotic versus inflammatory abdominal aortic aneurysm [10]. Mycotic aneurysms usually present as saccular aneurysm with irregular configuration, soft tissue stranding, and periaortic fluid or air collection. On the contrary, inflammatory aortic aneurysm presents as fusiform dilation and homogenous wall thickening with an increase in connective tissue (“Mantle sign”) indicative of sclerosing inflammation [10]. CT abdomen findings in our case were more suggestive of mycotic aneurysm with its irregular configuration and soft tissue stranding. Mycotic aneurysms are treated with IV antibiotics and open surgical or endovascular repair. On the contrary, an inflammatory aortic aneurysm requires aggressive immunosuppression. Immunosuppressive therapy can lead to rapid overgrowth of organisms, potential rupture, and ultimately be fatal if used in patients misdiagnosed with mycotic aneurysms [8, 9]. We initially did not treat this patient with corticosteroids as he did not have clinical and serological evidence of active EGPA, and his CT scan findings were more suggestive of a mycotic aneurysm. The C-reactive protein and procalcitonin levels could be high in both active vasculitis and sepsis, and one study suggested that a high procalcitonin level is more suggestive of infection rather than active vasculitis [11]. Our patient underwent an open surgical repair after the aneurysm rupture. Interestingly, the aortic biopsy findings were consistent with the diagnosis of EGPA vasculitis, and all the infectious disease workup was negative for any form of infection. Early institution of therapy would have prevented aneurysmal rupture, but we are not sure.

In summary, a diagnosis of inflammatory aortic aneurysm secondary to vasculitis should be considered in patients with a known diagnosis of EGPA, presenting with abdominal pain and having CT findings of aortic aneurysm. Early diagnosis and interventions are essential to avoid potential rupture. CT findings may not help distinguish the mycotic vs. inflammatory aneurysms. Our patient did not have any peripheral eosinophilia and had a negative ANCA at the time of presentation to the emergency department. This indicates that EGPA may not be active at the time of presentation, but no conclusion can be drawn based on one case report.

## Data Availability

All the data were included in the manuscript.

## Abbreviations

EGPA: Eosinophilic granulomatosis polyangiitis
CSS: Churg Strauss syndrome
ANCA: Antineutrophil cytoplasmic antibody
AAV: ANCA-associated vasculitis
ESR: Erythrocyte sedimentation rate
CRP: C-reactive protein
LFT: Liver function test
GGT: Gamma glutamyl transferase
ANA: Antinuclear antibody
c-ANCA: Cytoplasmic antineutrophilic antibodies
PR3: Proteinase3
MPO: Myeloperoxidase
p-ANCA: Perinuclear antineutrophil cytoplasmic antibodies
ED: Emergency department
PTFE: Polytetrafluoroethylene
PAS: Periodic acid Schiff stain
GPA: Granulomatosis polyangiitis
MPA: Microscopic polyangiitis.
